# Treatment Patterns and Outcomes in a Nationwide Cohort of Older and Younger Veterans with Waldenström Macroglobulinemia, 2006–2019

**DOI:** 10.3390/cancers13071708

**Published:** 2021-04-04

**Authors:** Hsu-Chih Chien, Deborah Morreall, Vikas Patil, Kelli M. Rasmussen, Christina Yong, Chunyang Li, Deborah G. Passey, Zachary Burningham, Brian C. Sauer, Ahmad S. Halwani

**Affiliations:** 1 George E. Wahlen Veterans Health Administration, Salt Lake City, UT 84148, USA; s66001028@gmail.com (H.-C.C.); u0058546@gcloud.utah.edu (D.M.); u6003644@gcloud.utah.edu (V.P.); kelli.rasmussen@hsc.utah.edu (K.M.R.); c.m.yong@utah.edu (C.Y.); u6017077@gcloud.utah.edu (C.L.); Deborah.Passey@hsc.utah.edu (D.G.P.); Zach.Burningham@hsc.utah.edu (Z.B.); brian.sauer@utah.edu (B.C.S.); 2 Division of Epidemiology, VERITAS, University of Utah, Salt Lake City, UT 84132, USA; 3 Division of Hematology and Hematologic Malignancies, Huntsman Cancer Institute, Salt Lake City, UT 84112, USA

**Keywords:** Waldenström macroglobulinemia, rare cancer, real-world evidence, older adults, treatment patterns, treatment outcomes, age groups

## Abstract

**Simple Summary:**

Waldenström macroglobulinemia is a rare cancer about which little is known. Evidence from real-world settings provides invaluable information to patients and clinicians, especially for older and/or frailer patients, a demographic often excluded from clinical trials. This study provides information about treatment patterns and outcomes from real-world cohorts of older (>70 years) and younger (≤70 years) patients. We report findings across early (2006–2012) and modern (2013–2019) eras, reflecting a transition during which the number of treatments available for Waldenström macroglobulinemia rapidly increased. We found marked improvements in treatment outcomes among older patients in the modern vs early era, with little or no improvement in outcomes among younger patients. Our findings emphasize the importance of real-world evidence in guiding patient-specific treatment decisions.

**Abstract:**

Little is known about real-world treatment patterns and outcomes in Waldenström macroglobulinemia (WM) following the recent introduction of newer treatments, especially among older adults. We describe patterns of first-line (1 L) WM treatment in early (2006–2012) and modern (2013–2019) eras and report outcomes (overall response rate (ORR), overall survival (OS), progression-free survival (PFS), and adverse event (AE)-related discontinuation) in younger (≤70 years) and older (>70 years) populations. We followed 166 younger and 152 older WM patients who received 1 L treatment between January 2006 and April 2019 in the Veterans Health Administration. Median follow-up was 43.5 months (range: 0.6–147.2 months). Compared to the early era, older patients in the modern era achieved improved ORRs (early: 63.8%, modern: 72.3%) and 41% lower risk of death/progression (hazard ratio (HR) for PFS: 0.59, 95% CI (confidence interval): 0.36–0.95), with little change in AE-related discontinuation between eras (HR: 0.82, 95% CI: 0.4–1.7). In younger patients, the AE-related discontinuation risk increased almost fourfold (HR: 3.9, 95% CI: 1.1–14), whereas treatment effects did not change between eras (HR for OS: 1.4, 95% CI: 0.66–2.8; HR for PFS: 1.1, 95% CI: 0.67–1.7). Marked improvements in survival among older adults accompanied a profound shift in 1 L treatment patterns for WM.

## 1. Introduction

Waldenström macroglobulinemia (WM) occurs in approximately three per million people per year, with 1400 new diagnoses in the USA annually [1,2]. The median age at diagnosis is 70 years [3,4].

Due to its rarity, little is known about WM treatment and outcomes. WM is incurable with currently available therapies, although not all patients require immediate treatment. Criteria for treatment initiation include immunoglobulin M (IgM)-related complications and/or symptoms related to direct bone marrow involvement by tumor cells [5]. Symptom severity dictates the intensity of the regimen chosen; patients with highly symptomatic disease are offered intense regimens with a potentially quicker response but increased side effects, whereas patients with minimal symptoms receive slower, less toxic regimens. Classical intense frontline treatments include alkylators (e.g., chlorambucil) and nucleoside analogues (e.g., fludarabine), either in combination with a monoclonal antibody (e.g., rituximab) or alone. Other classical frontline options include combination chemotherapies such as cyclophosphamide, doxorubicin, vincristine, and prednisone with rituximab (R-CHOP) [6]. Single-agent rituximab has long been considered safe for older/frailer patients because of its favorable toxicity profile [5]. Combined dexamethasone, rituximab, and cyclophosphamide (DRC) provide another option for older/frailer patients requiring active first-line combination therapy [7].

Emerging knowledge of relevant mutations has led to a modern era, beginning around 2013, of expanded treatment options for WM [6]. Resulting therapies, including bendamustine with rituximab (BR), bortezomib-based therapy, and ibrutinib, provide durable, effective options [7]. BR showed superior progression-free survival and tolerability compared to R-CHOP in a phase 3 clinical trial [8] and has been listed as a primary treatment option, especially for patients with high tumor bulk, by International Workshops on WM consensus since 2014 [9]. Phase 2 studies of active combinations of bortezomib with rituximab ± dexamethasone (BDR) showed partial response rates between 66% and 83%, with shortened response times (2–3 months) [10,11]. Neurotoxicity is a major concern with bortezomib-based regimens because underlying IgM-related neuropathy or neuropathies due to age-related comorbidities (such as diabetes) are common in WM patients [11,12]. Ibrutinib, an oral Bruton tyrosine kinase inhibitor, was the first therapeutic agent to receive US Food and Drug Administration (FDA) approval for the treatment of WM, in January, 2015 [13].

To our knowledge, there exist no studies that have quantified the changes in real-world WM treatment patterns that followed the introduction of newer therapies. The existing literature also lacks an understanding of real-world clinical outcomes after primary treatment for WM. Furthermore, there is a critical gap in our understanding of the emergence of novel therapies and their ability to improve treatment outcomes in older patients.

Thus, we sought to describe patterns of primary WM treatment from 2006 to 2019, relative to the introduction of newer therapies during the period of transition beginning in 2013. Using segmented linear regression models and data from the largest integrated health system in the US, the Veterans Health Administration (VHA), we estimated the impact of the publication of clinical trial outcomes and/or FDA approval of newer agents on the utilization of the most common first-line treatments for WM. We analyzed survival patterns of patients who started primary treatments in the early (2006–2013) and modern (2013–2019) eras, stratified by age and by time elapsed between diagnosis and primary treatment initiation. Finally, we presented occurrences of adverse event (AE)-driven discontinuation for primary treatments in both eras.

We hypothesized that novel therapies BR, BDR, and ibrutinib would supersede older treatments in the modern era, and that the introduction of ibrutinib would result in a decrease in the utilization of BR and BDR. Since single-agent rituximab has been used widely in older patients due to its mild toxicity, and BR and ibrutinib both show high tolerance in older/frail patients, we assumed that the publication of BR trial results and the FDA approval of ibrutinib would each decrease the utilization of single-agent rituximab. Lastly, given that the introduction of novel therapies profoundly broadened the spectrum of treatments available for older/frail patients, we hypothesized that older patients would demonstrate superior treatment outcomes in the modern vs the early era.

## 2. Materials and Methods

### 2.1. Cohort

**Figure 1:**
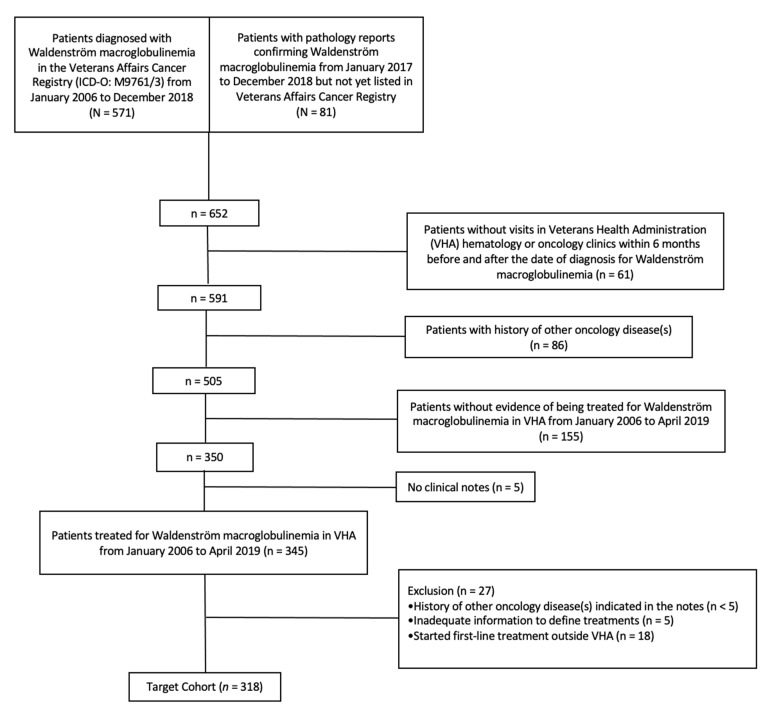
Cohort selection flowchart.

Combining information from the Veterans Affairs (VA) Cancer Registry System, pharmacy dispensation records, pathology reports, and clinical notes with a human chart review, we identified 345 patients diagnosed with WM who started first-line (1 L) therapy between January 1, 2006, and December 31, 2018, within the VHA. A human chart review of all hematology and oncology clinical notes was conducted to confirm patient eligibility and to exclude those patients with a history of another cancer diagnosis (*n* < 5), patients without adequate information to define treatments (*n* = 5), and those patients who received 1 L outside the VHA (*n* = 18). Of note, given the uncertainty regarding the impact of previous oncology treatments on treatment decision and outcomes in WM, we excluded patients with any history of another oncology disease (other than skin cancer) as documented in the VA Cancer Registry or clinical notes. We followed 318 eligible patients from 1 L treatment initiation date (confirmed by chart review) until loss to follow-up, death, or the end of the annotation period (30 June 2019) (see Figure 1 and Table 1). This study was approved by the University of Utah Institutional Review Board under IRB_00118129.

### 2.2. Patient and Disease Characteristics

We extracted patient demographics, disease features, patient comorbidities, and other WM-specific information from the VA Cancer Registry System and the VA Corporate Data Warehouse. The VA Cancer Registry System includes age, sex, race, vital status, and diagnosis date for all cancer cases diagnosed and/or treated in the VHA since 1995. Corporate Data Warehouse data consist of nationwide VHA clinical and administrative data systems that include structured data such as dispensation records and lab results, and unstructured electronic health records data such as clinical and pathology notes. VA Corporate Data Warehouse data refresh daily and are available from as early as October 1, 1999, to the present day. We extracted the US National Cancer Institute Comorbidity Index value (NCI index) [14] based on International Classification of Diseases (ICD-9-CM and ICD-10) diagnostic codes from inpatient and outpatient data within one year before 1 L initiation. WM-specific information included diagnosis date; average hemoglobulin level, platelet count, and IgM level within one year prior to 1 L; testing of biomarkers (*MYD88* and *CXCR4*); and hepatitis C screening before 1 L treatment.

#### Treatments and Patterns

Through the chart review, we identified 1 L treatment start and end dates and the type of 1 L treatments, defined using the following categories: (1) BR; (2) BDR; (3) ibrutinib; (4) single-agent rituximab; (5) DRC; (6) R-CHOP, chlorambucil-based, or fludarabine, cyclophosphamide, and rituximab (FCR); and (7) other (see Table 2).

Clinical notes and treatment dispensation records provided information for treatment start and end dates, between which patients were considered to have received active treatment. For patients treated with injectable therapeutics, the treatment end date was defined as the date of the last recorded dose of the regimen. For patients who received oral antineoplastic treatment, the treatment end date was defined as the medication run-out date, estimated using the date of the last dispensation. We calculated relative frequencies for the type of 1 L per quarter-year by assigning active treatments to quarter(s) based on start and end dates, and using the following equation:

(1) $$Relative\;frequency\;\left(\%\right)=\frac{Number\;of\;patients\;receiving\;a\;specific\;1L\;within\;the\;quarter}{Number\;of\;patients\;receiving\;any\;active\;1L\;within\;the\;quarter}$$

### 2.3. Clinical Outcomes

We defined early (2012 and before) and modern (2013 and after) treatment eras. The date a patient’s 1 L treatment was initiated determined the assigned treatment era. Clinical outcomes included treatment responses, overall survival (OS), progression-free survival (PFS), and AE-related discontinuation of 1 L. OS, PFS, and the time to AE-related discontinuation were calculated from the date of 1 L initiation. We retrieved dates of death from VA Corporate Data Warehouse records, whereas dates of disease progression and/or AE-related discontinuation were established through human annotation of clinical notes. Only cases with clearly mentioned dates of disease progression and/or AE-related discontinuation were included in the analysis. Clinicians’ impressions served as primary sources for treatment responses, with changes in serum IgM level from baseline (according to International Workshops on WM response criteria [9]) as a secondary source. Per the FDA’s PFS censoring scheme recommendation to consider death between two assessment visits (every 90 days within VA) as an event for PFS, we considered both disease progression and death within 90 days of the last hospital visit as events for PFS estimation in the primary analysis [15].

### 2.4. Treatment Pattern Analyses

We applied an interrupted time series (ITS) design with a segmented regression model to describe treatment pattern changes quantitively [16]. We defined a transitional time period which included several key events in WM treatment, then compared linear trends of pre- and post-transition monthly relative frequencies [17] with generalized estimating equations [18], evaluating BDR, BR, ibrutinib, DRC, and single-agent rituximab.

We defined the time period from February 2013 to January 2015 (inclusive) as transitional because this period encompassed multiple milestone events for WM treatment, including: (1) the publication of phase 3 clinical trial results of BR in February 2013 [8], (2) the announcement of updated guidelines by the European Society for Medical Oncology [19] in October 2013 and the Seventh International Workshops on WM [9] in July 2014, and (3) the FDA approval of ibrutinib for WM in January 2015 [13]. We did not set out to analyze the impact of each milestone event individually and, furthermore, obtaining such an estimate would not be possible with events occurring so close together. Thus, we defined the time period from February 2013 to January 2015 as transitional and did not include any observations from the transition. This design is intended to facilitate an accurate evaluation of the net effects of the entire transition on treatment pattern trends.

For the trend change evaluation, the pre-transition period began when the regular use of a specific regimen was established. We defined the start of pre-transition as the later of either the first publication of phase 2 clinical trials results (if any) or initial prescription in a patient. Within the VHA, prescription of a newer or uncommonly used regimen must typically be justified with clinical evidence; thus, we considered the publication date of first phase 2 clinical trial results to be a reasonable start date for the transition period. In the absence of such results, or if results were published before the beginning of the study data, the date of initial prescription served as the start date for the pre-transition period (see Table 3). The post-transition period started at the end of the transition period (i.e., post-transition started in February 2015), and ended in June 2019.

### 2.5. Outcome Analyses

We stratified patients by age (70 years old and younger vs older than 70 years), with estimates serving as characteristic-based controls [22]. Table 4 provides additional analytic results that model effects separately.

Survival was estimated using the Kaplan–Meier method, and comparisons were made by log-rank tests. Cox proportional-hazards regression models were used to evaluate treatment benefits in OS and PFS. A cause-specific Cox model was applied to estimate the risk of AE-driven discontinuation. Patients lost to follow-up were censored from the date when last known to be alive. To present differences in baseline characteristics between groups, we reported estimates of effects and their 95% confidence intervals (95% CIs). All statistical analyses were completed using R 3.5.3.

#### Subgroup and Sensitivity Analyses

Patients who were symptomatic and thus required immediate treatment at diagnosis might have a different disease activity status from those who were asymptomatic and had a longer watch-and-wait duration. We thus conducted a subgroup analysis focusing on patients who started 1 L within 3 months of diagnosis to determine how disease status at 1 L initiation might impact outcome estimates of OS, PFS, and discontinuation due to AE.

We performed 2 sensitivity analyses to check the robustness of the PFS estimates in extreme settings. First, we assumed that only cases of disease progression were those with “true events.” Death after the last visit was not included. Time to progression was applied in a Cox cause-specific model for hazard ratio (HR) estimation. Second, we assumed that death at any point after the last visit was associated with WM progression, yielding the most conservative estimate of PFS. Similar point estimates and overlapping 95% CIs in primary and sensitivity analyses demonstrated the robustness of our analytical methods and findings.

## 3. Results

Among 318 confirmed diagnosis of WM, 138 patients initiated 1 L in 2012 or before, with 180 beginning 1 L in 2013 or after. The mean age at diagnosis was 69.3 years (standard deviation [SD]: 9.5 years), with 145 patients (45.6%) being older than 70 years of age at diagnosis. Patient characteristics of younger and older WM patients by the era of 1 L treatment are shown in Table 1.

### 3.1. Patient Characteristics

Other than in age and comorbidity status, demographic and disease-specific features did not change in younger patients across eras. Younger patients who started 1 L in the modern era were older than those who started 1 L in the early era (early: 61.4 years, SD: 6.3 years; modern: 64.0 years, SD: 5.5 years; *p* = 0.01). A higher percentage of younger patients who started 1 L in the modern era had an NCI index ≥2 (early era: 23.9%; modern era: 27.8%). Among older patients, patients who started 1 L in the modern era did not significantly differ in age at 1 L initiation from those who started 1 L in the early era (early: 78.6 years, SD: 5.5 years; modern: 77.0 years, SD: 5.7 years; *p* = *0*.07). Older patients in the modern era had more comorbidities (NCI index ≥2 in early era: 31.7%; modern era: 43.8%), with a lower average IgM value before starting 1 L (median IgM in early era: 3932 mg/dL; modern era: 2556 mg/dL). The percentages of patients starting 1 L within 3 months of diagnosis were similar in the younger populations (early era: 66.7%, modern era: 67.0%) but were higher in older patients of the early era (early era: 74.6%, modern era: 65.2%). All patients who received 1 L treatments were tested with hemoglobulin and platelets before starting 1 L. A small proportion (16.4%) of patients did not receive IgM testing, with no significant difference in rates of testing between eras. Low screening rates for *MYD88* and *CXCR4* were observed in both younger and older populations. The number of patients tested for *MYD88* in the early era were too low to report; in the modern era, 23.1% of younger patients and 21.3% of older patients received *MYD88* testing. The numbers of patients receiving *CXCR4* screening in both eras were too low to report.

#### Treatment Patterns

**Figure 2:**
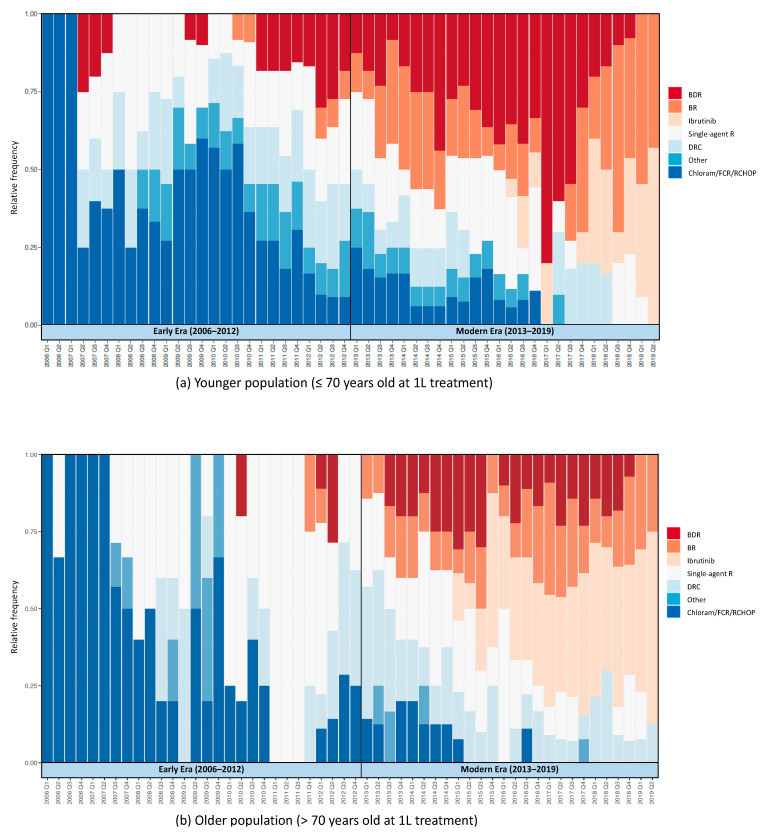
1 L treatment patterns in veterans with Waldenström macroglobulinemia, 2006–2019: (**a**) 1 L treatment pattern in younger patients (≤70 years of age at initiation of 1 L therapy); (**b**) 1 L treatment pattern in older patients (>70 years of age at initiation of 1 L therapy).1 L: first-line treatment; BDR: bortezomib and dexamethasone +/− rituximab; BR: bendamustine +/− rituximab; Chloram: chlorambucil; DRC: dexamethasone, rituximab, and cyclophosphamide; FCR: fludarabine and cyclophosphamide +/− rituximab; R: rituximab; RCHOP: cyclophosphamide, doxorubicin, vincristine, and prednisone ± rituximab.

Figure 2a,b show 1 L treatment patterns in Veterans with WM from 2006 to 2019. Preferred 1 L therapies shifted from alkylator- and nucleoside analogue-based regimens and rituximab monotherapy to bendamustine-based and targeted therapy-based regimens with time. The number of patients per quarter for each year are shown in Appendix AFigure A1. Overall, single-agent rituximab was the most commonly used 1 L treatment from 2006 to 2019. We also observed that the introduction of a new therapy in older patients always occurred either within the same quarter as the first publication of phase 2 clinical trial results or after, whereas younger patients tended to receive a new therapy ahead of result publication, except for ibrutinib.

**Figure 3:**
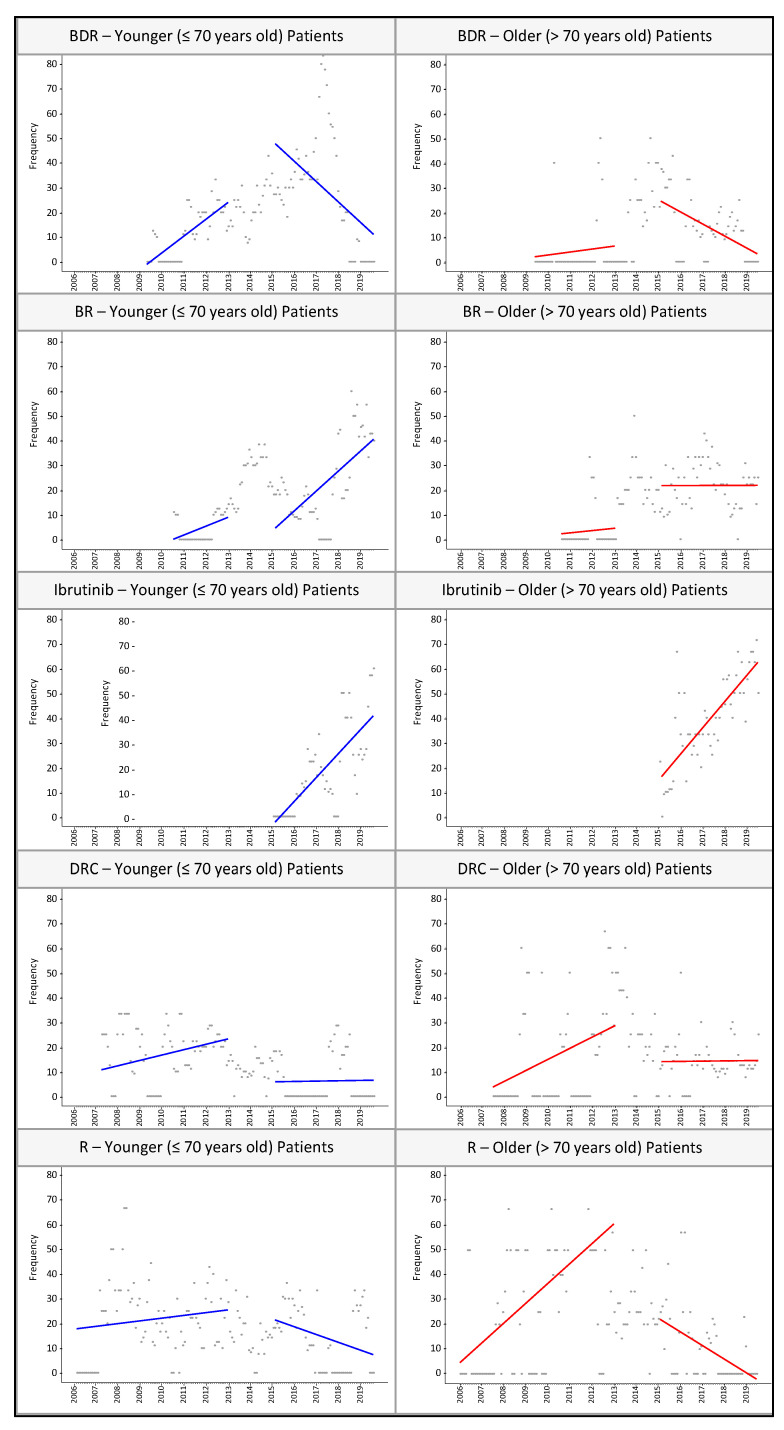
Treatment utilization trends in BDR, BR, ibrutinib, DCR, and single-agent rituximab, 2006–2019. BDR: bortezomib and dexamethasone +/− rituximab; BR: bendamustine +/− rituximab; DRC: dexamethasone, rituximab, and cyclophosphamide; R: rituximab.

Figure 3 illustrates trends in BDR, BR, ibrutinib, DRC, and single-agent rituximab in pre- and post-transition periods. After the transition, BR saw increasing use, with no significant change in DRC and decreased use of BDR and single-agent rituximab. The post-transition decline in BDR use was more precipitous among younger patients (change in slope for BDR use in younger patients: −1.29, 95% CI: −1.75 to −0.82; older patients: −0.51, 95% CI: −0.7 to −0.32). Older patients had a more drastic negative change in the use of single-agent rituximab (change in slope in younger population: −0.37, 95% CI: −0.65 to −0.08; older: −1.14, 95% CI: −1.61 to −0.67). Ibrutinib use increased profoundly in both populations after its approval for WM; in older patients, it was adopted within the same month of its approval, whereas its initial utilization in the younger population occurred approximately 8 months after approval.

### 3.2. Treatment Outcomes

Table 5 provides treatment outcomes in early and modern eras. Older patients receiving 1 L within the modern era showed higher ORRs (early: 63.8%, modern: 72.3%), whereas younger patients achieved similar ORRs in both eras (early: 75.0%, modern: 75.9%). Median OS among all older patients was 68.5 months (95% CI: 55.5–102.6 months). Median OS among all younger patients was 109.2 months (95% CI: 94.3–(not reached)). Median OS was 55.5 months (95% CI: 31.8–92.1 months) in older patients in the early era, and 122.4 months (95% CI: 100.9 months–(not reached)) in younger patients in the early era; median OS was not reached in either the younger or older population in the modern era. Among older patients, median PFS improved drastically from 28.3 months (95% CI: 18.5–55.6 months) in the early era to 63.3 months (95% CI: 32.0 months–(not reached)) in the modern era. Younger patients, on the other hand, showed almost no improvement in median PFS: Median PFS among younger patients in the early era was 52.7 months (95% CI: 43.1–97.4 months), and in the modern era it was 52.8 months (95% CI: 41.2–(not reached)).

A higher proportion of younger patients in the modern era experienced AE-related treatment discontinuations (early: 4.0%; modern: 14.3%, *p* = *0*.03), whereas proportions were similar between eras in the older population (early: 22.2%; modern: 18.0%, *p* = *0*.52) (Table 6).

**Figure 4:**
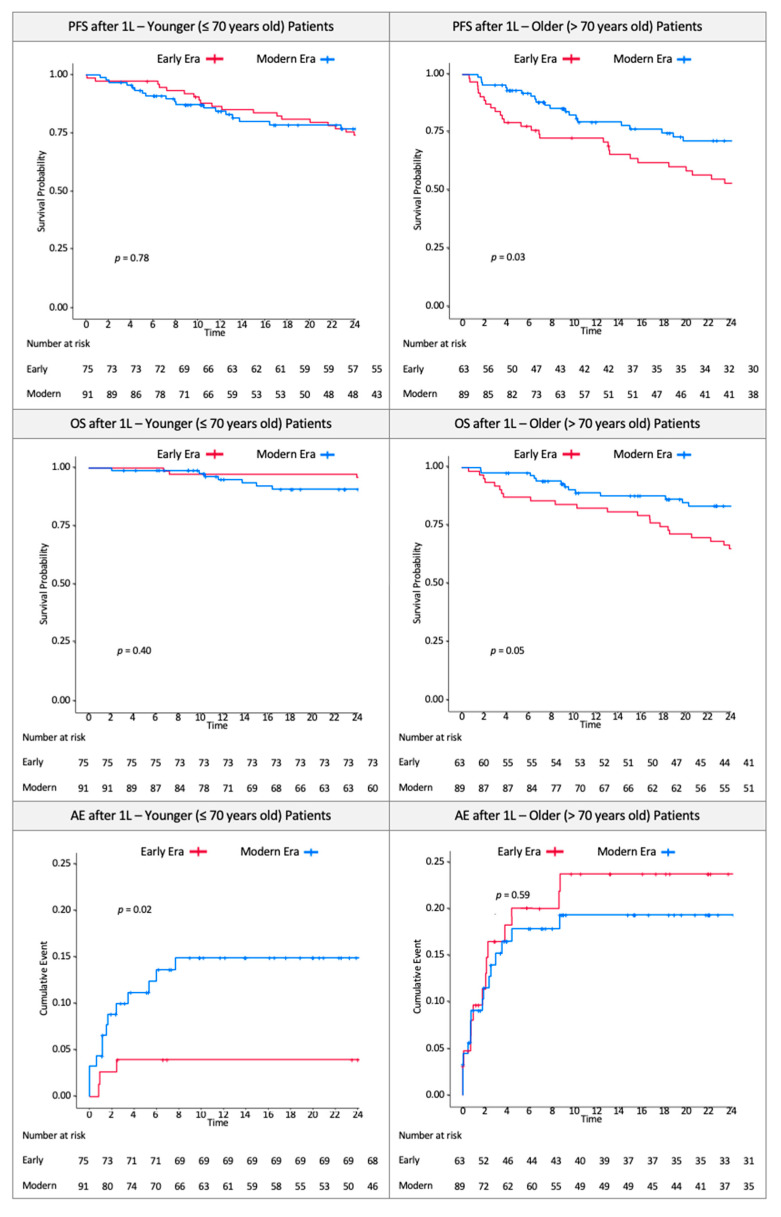
Progression-free survival, overall survival, and adverse event-related discontinuation in veterans with Waldenström macroglobulinemia, 2006 to 2019. 1 L: first-line treatment; AE: adverse event; Early: early era, 2006–2012; Modern: modern era, 2013–2019; OS: overall survival; PFS: progression-free survival.

**Figure A2:**
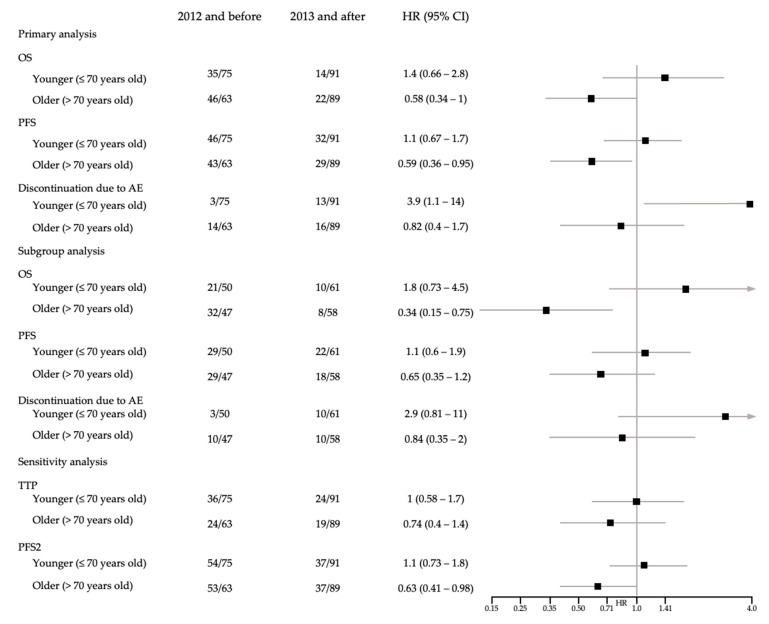
Hazard ratios of overall survival, progression-free survival and discontinuation due to adverse events in patients receiving first-line treatments for Waldenström macroglobulinemia in early (2006 to 2012) and modern eras of younger (70 years of age or younger at initiation of 1 L therapy) and older (older than 70 years of age at initiation of 1 L therapy) populations, in primary and sensitivity analysis settings. AE: adverse event; CI: confidence interval; HR: hazard ratio; PFS: progression free survival; PFS2: progression free survival counting all deaths after lost of follow up as events for PFS (the second setting for sensitivity analyses); OS: overall survival; TTP: time to progression (the first setting of sensitivity analyses).

Figure 4 illustrates curves for OS, PFS, and time to AE-related discontinuation. Treatment effect estimates with OS (HR: 1.4, 95% CI: 0.66–2.8) and PFS (HR: 1.1, 95% CI: 0.67–1.7) were similar in younger patients in the early vs the modern era, whereas the older patients demonstrated improvements in OS and PFS. The risk of AE-related discontinuation increased in younger patients in the modern era by 3.9 times (HR: 3.9, 95% CI: 1.1–14), whereas the risk among older patients remained similar across eras (HR: 0.82, 95% CI: 0.4–1.7). Subgroup and sensitivity analyses yielded similar results (Appendix A Figure A2).

## 4. Discussion

Because of the rarity of WM, clinical evidence for the primary treatment of WM is limited. Treatments have been adopted based on data derived from phase 2 studies or from randomized studies of which the populations included not only WM but also other indolent B-cell malignancies. In two published phase 3 clinical trials, participants either had better performance status [23] or were younger [24] than the typical real-world patient with WM. A recent study reported real-world evidence from a population of only younger patients (median age 49 years) [25]; another study reported real-world evidence from a cohort with 1 L initiation dates through the end of 2013 (prior to the introduction of ibrutinib) [26]. In this study, we report treatment responses, OS, PFS, and discontinuation rates in younger and older populations that received 1 L treatment in early (2006 to 2012) and modern (2013 to 2019) eras. Our rigorous chart review process included internal data quality control measures and cross-validation of results with structured electronic health records. Furthermore, we performed subgroup and sensitivity analyses to confirm the robustness of our findings, yielding high-quality clinical evidence for WM treatments from real-world settings. Our study of modern treatments [3,4] in a real-world setting represents the largest cohort study of modern primary WM treatment to date.

To our knowledge, this study is the first to report treatment response rates to WM therapies in a real-world setting including both older and younger patients [25]. We found ORRs of 76% (younger patients) and 69% (older patients), both lower than an aggregated ORR based on clinical trials of rituximab-based combination therapy (ORR: 84%, 95% CI: 81–87%) [27].

We are also the first to report the median OS and median PFS in younger and older patients after 1 L WM treatments in a real-world setting. Previous studies of WM in real-world settings either reported median OS from a WM population of both treated and untreated individuals [4] or did not report the median OS because it was not achieved [26,28]. We found that the median PFS estimate among all older patients was 36.9 months (95% CI: 29.3–63.3 months) and among all younger patients was 52.7 months (95% CI: 43.5–94.3 months). The median PFS estimates in clinical trials were 20.3 months (single-agent R) [23], 42 months (BDR) [12], and 34 months (DRC) [29]; the median PFS was not achieved for BR [30] and ibrutinib [23]. In clinical trials, the median OS was not achieved.

This report is the first, to our knowledge, to describe patterns of lab and biomarker testing rates in a cohort including both younger and older patients in a modern, real-world setting [25,26,28]. All patients who received 1 L treatments were tested with hemoglobulin and platelets before 1 L. About one-sixth (16.4%) did not receive IgM testing, with no significant difference observed in rates of testing in the early vs modern era. It is not surprising that not all treated patients had an IgM value before 1 L, since treatment indications were based on clinical or laboratory findings [17,30]. *MYD88* mutation status, a predictor for survival and treatment response [31], was initially tested in the VHA in 2015. However, despite being recommended as an essential workup in the 2016 National Comprehensive Cancer Network Guidelines [32], less than a quarter of patients in our cohort received *MYD88* screening before 1 L.

Our ITS analysis quantified how treatment patterns changed after a transitional period of published clinical trial results, updated guidelines, and the first and only FDA approval of a WM treatment (ibrutinib). We observed treatment pattern changes in older patients, with a larger decrease in single-agent rituximab use and a smaller decrease in BDR use, compared to younger patients.

Clinical outcomes for younger and older patients differed in several respects. In older patients, we observed an increase in survival and fewer AE-associated discontinuations accompanying their treatment pattern changes. We consider the reduced risk of death, disease progression, and AE-related discontinuation among older patients a natural consequence of the introduction of low-toxicity treatments with better treatment effects than single-agent rituximab [29]. We also observed that, among older patients, those who started 1 L in the modern era presented lower IgM values at baseline. This finding may simply illustrate the heterogeneity of WM, given that IgM has been considered neither a laboratory indication in treatment consensus [9,24] nor a prognostic factor for WM [24,28].

Although older patients achieved improved outcomes (ORR, OS, and PFS) in the modern vs early era, little or no change was observed in clinical outcomes in younger patients. A real-world study of a cohort of younger (median age 49) patients in Italy observed an ORR (81%) similar to the ORR in the younger population in our study (75%), and also found no improvement in OS in later (2010–2019) vs earlier (diagnosed 2000–2009) eras, similar to our findings [25]. Differences in OS and PFS estimates in younger patients could reflect the impact of age (younger patients who started 1 L in the modern era were 2.6 years older than those who started 1 L in the early era). Our findings of greater improvement in older patients may reflect the fact that all intensive treatments available before 2013 bore significant toxicities, with limited options for older/frail patients. Novel therapies’ main advantage lay in improved tolerability, so older (typically frailer) patients received greater benefits from the introduction of effective, low-toxicity medications than younger, less frail patients who had continued access to more aggressive treatment options.

Our findings demonstrate the rapid adoption of evidence-based treatment of WM in older patients within the VHA. The initial utilization of DRC occurred within the same quarter as the publication of its phase 2 clinical trial [20]. After the publication of BR (phase 3) [8] and BDR (phase 2) [12] clinical trial results, increased utilization of those therapies quickly followed. Our results also show the rapid adoption of novel agent ibrutinib, which was first utilized in the older population within the same month of FDA approval. In contrast, younger patients tended to receive newer treatments before evidence from clinical trials was established, the exception being the use of ibrutinib, which was not observed in younger patients until 8 months after FDA approval. We hypothesize that providers may have viewed ibrutinib as insufficiently aggressive for younger, less frail patients.

The tendency for younger patients to receive new therapies before the publication of clinical trial results may explain the higher risk of AE-related discontinuation among younger patients. Older patients who waited to try new therapies could have benefited from dosage, safety/efficacy, and other supportive information provided in the clinical trial results. Further research to determine the impact of evidence-based practice and their clinical outcomes will help foster treatment benefits with improved quality of care.

### Limitations

The findings reported in this study are derived from VHA data, and thus may not be generalizable to other populations. However, the disease- and patient-specific characteristics of the study population reflect those of the general population of patients with WM (with the exception that the VHA patient population is mostly male), and basing our analysis on data from patients within the same care system allowed us to achieve our goal of comparing outcomes of treatments given in the early vs modern era. Our study relied on the use of VHA clinical notes to identify treatments, suggesting that we may have missed a treatment or procedure that occurred outside the VHA. Any treatments received outside the VHA that were commented on or documented by VHA providers in their clinical notes were extracted through human annotation, therefore we believe we have captured an accurate and complete treatment history for our study population. Even so, in an effort to mitigate any potential under-capture of disease progression due to care received outside the VHA or death after the last follow-up, we performed the two sensitivity analyses described previously in the Materials and Methods section, which confirmed the robustness of our analytical methods and findings.

We applied an ITS design with a segmented regression model to estimate quantitative changes in treatment patterns before and after a transition period, which included milestone events. Our results cannot distinguish the impact of individual events, nor establish causality between the transition and treatment changes. Causality with an ITS design assumes that no other events could have impacted treatment pattern changes across the pre- and post-transition periods, such that patterns would have remained the same in a counterfactual condition. This assumption can be considered a strong assumption if we are unsure as to whether any other events could have impacted treatment patterns. In addition, although stability was achieved with 24 time points in the model, the estimates of changes in slope could have been underpowered due to limited case numbers per time point [33]. Nonetheless, the results of an ITS design with a segmented regression model still demonstrate quantitatively how a trend changes before and after a transition.

We captured only AEs which led to treatment discontinuation; thus, our findings cannot be compared with AE data from clinical trials. Additionally, limited case numbers could call into question the power of our risk estimates for AE-related discontinuation in older patients. However, we are confident in the risk estimates because of our extremely meticulous chart review to confirm cases, combined with the fact that accurate outcome measurements yield unbiased risk estimates.

Very few patients in either cohort received *MYD88* screening in the early era. Therefore, it is possible that patients with other lymphomas could have been misdiagnosed as having WM and mistakenly included in the study cohort. Although we believe that the numbers of such potentially misdiagnosed patients (if any) would be small, and would not likely skew our analyses, there is always the possibility that a sufficient number of misdiagnosed patients could have been included in the cohort.

Lastly, our findings do not support the conclusion that the introduction of newer therapies alone accounts for treatment benefits in the modern era. Advances in other modes of management, such as autologous stem cell transplantation and plasmapheresis, and quality of care, occurred within a similar time frame and could also contribute to these results. However, our findings are free from lead-time bias, given that indications for treatment initiation did not change within the observation periods [5,9,21,27]. Comparative effectiveness and safety studies on older versus newer therapies that adjust for factors potentially associated with outcomes are needed to provide further data to facilitate clinicians’ and patients’ informed decision-making.

## 5. Conclusions

The landscape of WM primary treatments has changed profoundly with the introduction of newer therapies. Older patients especially demonstrated improvements in survival and AE-associated discontinuation rates accompanying the treatment pattern changes. Further comparative effectiveness and safety studies on different classes of treatments are warranted.

## Figures and Tables

**Table 1:** Characteristics in veterans receiving treatment for Waldenström macroglobulinemia in Veterans Affairs (VA) from 2006 to 2019.

Characteristics	Younger Population (<70 Years of Age at 1 L Treatment) (*n* = 166)				Older Population (>70 Years of Age at 1 L Treatment) (*n* = 152)			
	2006–2019 (*n* = 166)	2006–2012 (*n* = 75)	2013–2019 (*n* = 91)	*p*-Value	2006–2019 (*n* = 152)	2006–2012 (*n* = 63)	2013–2019 (*n* = 89)	*p*-Value
Age at 1 L								
Mean (SD)	62.8 (6.0)	61.4 (6.3)	64.0 (5.5)	0.01	77.7 (5.7)	78.6 (5.5)	77.0 (5.7)	0.07
Age at diagnosis								
Mean (SD)	62.4 (6.0)	61.0 (6.3)	63.2 (5.6)	0.02	77.0 (5.8)	78.3 (5.7)	76.1 (5.8)	0.02
>70 years of age no. (%)	–	–	–		145 (95.4)	61 (96.8)	84 (94.4)	0.75
Male sex, no. (%)	162 (97.5)	73 (97.3)	89 (97.8)	1.0	150 (98.7)	62 (98.4)	88 (98.9)	1.0
Race, no. (%)								
Non-Hispanic White	134 (80.7)	62 (82.7)	72 (79.1)	0.69	137 (90.1)	56 (88.9)	81 (91.0)	0.93
Black	25 (15.1)	10 (13.3)	15 (16.5)		10 (6.6)	5 (7.9)	5 (5.6)	
Other	7 (4.2)	<5	<5		5 (3.3)	<5	<5	
Residential community, no. (%)								
Rural/Suburban	34 (20.5)	15 (20.0)	19 (20.9)	0.92	32 (21.1)	17 (27.0)	15 (16.9)	0.05
Metropolitan	132 (79.5)	60 (80.0)	72 (79.1)		120 (78.9)	46 (73.0)	74 (83.1)	
Residential geographic region, no. (%)								
Midwest	45 (27.1)	20 (26.7)	25 (27.5)	0.97	42 (27.6)	16 (25.4)	26 (29.2)	0.67
Northeast	28 (16.9)	12 (16.0)	16 (17.6)		24 (15.8)	12 (19.0)	12 (13.5)	
South	56 (33.7)	25 (33.3)	31 (34.1)		41 (27.0)	19 (30.2)	22 (24.7)	
West	37 (22.3)	18 (24.0)	19 (20.9)		41 (27.0)	14 (22.2)	27 (30.3)	
BMI ≥ 35 kg/m^2^, no. (%)	12 (7.2)	5 (6.7)	7 (7.7)	1.0	11 (7.2)	<5	9 (10.1)	0.19
NCI index at 1 L, no. (%)								
0	77 (46.4)	36 (48.0)	41 (45.1)	0.64	56 (36.8)	28 (31.5)	28 (31.5)	0.17
1	42 (25.3)	18 (24.0)	24 (26.4)		42 (27.6)	21 (23.6)	21 (23.6)	
≥2	42 (25.3)	17 (23.9)	25 (27.8)		59 (38.8)	20 (31.7)	39 (43.8)	
Not available, no. (%)	5 (3.0)	<5	<5		<5	0 (0)	<5	
Laboratory values within a year prior to 1 L								
Hemoglobin, g/dL								
Median (range)	10.8 (5.8–17.3)	11.2 (6.8–17.3)	10.6 (5.8–16.2)	0.28	9.9 (5.9–15.1)	9.7 (6.9–13.9)	10.1 (5.9–15.1)	0.88
Below LRL, no. (%)	142 (85.5)	63 (84.0)	79 (86.8)	0.79	137 (90.1)	58 (92.1)	79 (88.8)	0.61
Platelet count, 10^9^/L								
Median (range)	219.5 (26.5–866.3)	226.2 (26.5–866.3)	215.7 (33.1–451.5)	0.68	191.2 (11.1–586.7)	192.4 (11.1–586.7)	186.6 (26.5–503.0)	1.0
Below LRL, no. (%)	38 (22.9)	18 (24.0)	20 (22.0)	0.90	61 (33.6)	23 (36.5)	28 (31.5)	0.67
IgM, mg/dL								
Median (range)	3617 (16–9270)	3740 (229–8200)	3587 (16–9270)	0.50	3085.0 (10–9944)	3932 (10–9944)	2556 (84–9740)	0.08
Above URL, no. (%)	136/141 (96.4)	62/64 (96.9)	74/77 (96.1)	1.0	123/125 (98.4)	48 (76.2)	75 (84.3)	1.0
Not available, no. (%)	25 (15.1)	11 (14.7)	14 (15.4)	1.0	27 (17.8)	14 (22.2)	13 (14.6)	0.32
*MYD88*, no. (%)								
Tested	21 (12.7)	0 (0)	21 (23.1)	< 0.01	19 (12.5)	0 (0)	19 (21.3)	< 0.01
Wild type	6 (28.6)	–	6 (28.6)		< 5	–	< 5	
Mutation	10 (47.6)	–	10 (47.6)		16 (84.2)	–	16 (84.2)	
Results not available	5 (23.8)	–	5 (23.8)		< 5	–	< 5	
Hepatitis C Virus, no. (%)								
Tested	40 (24.1)	13 (17.3)	27 (29.7)	0.1	21 (13.8)	8 (12.7)	13 (14.6)	0.92
Positive	4 (10.0)	<5	<5		0 (0)	0 (0)	0 (0)	
Negative	17 (42.5)	6 (46.2)	11 (40.7)		14 (66.7)	<5	10 (76.9)	
Results not available	19 (47.5)	<5	15 (55.6)		7 (33.3)	<5	<5	
Time from diagnosis to 1 L								
Median, months (range)	1.3 (0.0–99.4)	1.2 (0.0–52.3)	1.4 (0.1–99.4)	0.42	1.2 (0.0–113.0)	0.7 (0.0–65.7)	1.6 (0.0–113.0)	<0.01
≥3 months, no. (%)	111 (66.9)	50 (66.7)	61 (67.0)	0.20	105 (69.1)	47 (74.6)	58 (65.2)	0.34
1 L Treatment, no. (%)								
BR	24 (14.5)	<5	22 (24.2)	<0.01	16 (10.5)	<5	15 (16.9)	<0.01
BDR	31 (18.7)	8 (10.7)	23 (25.3)		17 (11.2)	<5	14 (15.7)	
Ibrutinib +/− R	8 (4.8)	0 (0)	8 (8.8)		17 (11.2)	0 (0)	17 (19.1)	
Single-agent R	46 (27.7)	23 (30.7)	23 (25.3)		52 (34.2)	27 (42.9)	25 (28.1)	
DRC	19 (11.4)	11 (14.7)	8 (8.8)		19 (12.5)	8 (12.7)	11 (12.4)	
Chloram/FCR/R-CHOP	31 (18.7)	26 (34.7)	5 (5.5)		23 (15.1)	20 (31.7)	<5	
Other	7 (4.2)	5 (6.7)	<5		8 (5.3)	<5	<5	
Duration of 1 L								
Median, months (range)	3.5 (0.0–41.5)	3.6 (0.0–39.7)	3.1 (0.0–41.5)	0.31	1.9 (0.0–35.9)	1.8 (0.0–23.4)	2.3 (0.0–35.9)	0.27

1 L: first-line treatment; BDR: bortezomib and dexamethasone +/− rituximab; BMI: body mass index; BR: bendamustine +/− rituximab; Chloram: chlorambucil; CI: confidence interval; DRC: dexamethasone, rituximab, and cyclophosphamide; FCR: fludarabine and cyclophosphamide +/− rituximab; IgM: immunoglobulin M; LRL: lower reference limit; no.: number; NA: not available; NCI: National Cancer Institute; OS: overall survival; PFS: progression-free survival; R: rituximab; R-CHOP: cyclophosphamide, doxorubicin, vincristine, and prednisone ± rituximab; SD: standard deviation; URL: upper reference limit; VA: Department of Veterans Affairs.

**Table 2:** Treatment regimens received by Veterans with Waldenström macroglobulinemia, 2006 to 2019.

Regimen Category	Treatment Regimen	Number of Patients Treated
BR	Bendamustine monotherapy	<5
	Bendamustine and rituximab	39
BDR	Bortezomib monotherapy	<5
	Bortezomib and dexamethasone	6
	Bortezomib and rituximab	5
	Bortezomib, dexamethasone, and rituximab	35
Chloram/FCR/R-CHOP	Chlorambucil	19
	Chlorambucil and rituximab	<5
	Cyclophosphamide, vincristine, and prednisone with rituximab	11
	Cyclophosphamide, doxorubicin, vincristine, and prednisone with rituximab	6
	Cyclophosphamide, vincristine, and prednisone	<5
	Fludarabine, cyclophosphamide, and rituximab	<5
	Fludarabine monotherapy	<5
	Fludarabine and rituximab	11
DRC	Dexamethasone, cyclophosphamide, and rituximab	38
Ibrutinib +/− R	Ibrutinib	23
	Ibrutinib and rituximab	<5
Others	Bortezomib, cyclophosphamide, and dexamethasone	<5
	Bortezomib, cyclophosphamide, dexamethasone, and rituximab	<5
	Cladribine and rituximab	<5
	Carfilzomib, dexamethasone, and rituximab	<5
	Cyclophosphamide, melphalan, and rituximab	<5
	Cyclophosphamide monotherapy	<5
	Lenalidomide, bortezomib, and dexamethasone with rituximab	<5
	Thalidomide and dexamethasone	<5
	Thalidomide and rituximab	<5
Single-agent R	Rituximab and dexamethasone	<5
	Rituximab monotherapy	96

BDR: bortezomib and dexamethasone +/− rituximab; BR: bendamustine +/− rituximab; Chloram: chlorambucil; DRC: dexamethasone, rituximab, and cyclophosphamide; FCR: fludarabine and cyclophosphamide +/− rituximab; R: rituximab; R-CHOP: cyclophosphamide, doxorubicin, vincristine, and prednisone ± rituximab.

**Table 3:** Example milestones used to determine pre-transition start dates for ITS analysis.

Treatment Regimen	Date of Publication of First Phase 2 Clinical Trial	Date of Initial Prescription in VA	Start Date of Pre-transition Period
BR	NA	August 2010	August 2010
BDR	June 2009 [12]	June 2007	June 2009
DRC	June 2007 [20]	June 2007	June 2007
Single-agent R	May 2002 [21]	January 2006	January 2006

BDR: bortezomib and dexamethasone +/− rituximab; BR: bendamustine +/− rituximab; DRC: dexamethasone, rituximab, and cyclophosphamide; ITS: interrupted time series; NA: not applicable; R: rituximab; VA: Department of Veterans Affairs.

**Table 4:** Segmented regression analysis.

Treatment Regimen		Coefficient Estimates (95% CI)	
	Pre-Transition Slope	Post-Transition Slope	Change in Slope
**BDR**			
Younger	0.58 (0.21 to 0.95)	−0.67 (−0.92 to −0.42)	−1.29 (−1.75 to −0.82)
Older	0.10 (0.02 to 0.18)	−0.41 (−0.59 to −0.22)	−0.51 (−0.70 to −0.32)
**BR**			
Younger	0.31 (−0.16 to 0.77)	0.69 (0.46 to 0.92)	0.38 (−0.11 to 0.88)
Older	0.08 (−0.31 to 0.47)	0.0 (−0.11 to 0.11)	−0.08 (−0.50 to 0.35)
**Ibrutinib +/− R**			
Younger	–	0.83 (0.61 to 1.04)	–
Older	–	0.89 (0.69 to 1.10)	–
**DRC**			
Younger	0.19 (0.09 to 0.28)	0.01 (−0.16 to 0.19)	−0.17 (−0.38 to 0.03)
Older	0.38 (0.23 to 0.52)	0.06 (−0.06 to 0.18)	−0.37 (−0.69 to −0.05)
**Single-agent R**			
Younger	0.10 (−0.04 to 0.23)	−0.27 (−0.49 to −0.05)	−0.37 (−0.65 to −0.08)
Older	0.68 (0.47 to 0.89)	−0.46 (−0.69 to −0.24)	−1.14 (−1.61 to −0.67)

BDR: bortezomib and dexamethasone +/− rituximab; BR: bendamustine +/− rituximab; CI: confidence interval; DRC: dexamethasone, rituximab, and cyclophosphamide; FCR: fludarabine and cyclophosphamide +/− rituximab; R: rituximab; R-CHOP: cyclophosphamide, doxorubicin, vincristine, and prednisone ± rituximab; Younger: patients 70 years of age or younger at 1 L treatment; Older: patients older than 70 years of age at 1 L treatment.

**Table 5:** Outcomes in veterans receiving treatment for Waldenström macroglobulinemia in VA from 2006 to 2019.

Clinical Outcomes	Younger Population (<70 Years of Age at 1 L Treatment) (*n* = 166)			Older Population (>70 Years of Age at 1 L Treatment) (*n* = 152)		
	2006–2019 (*n* = 166)	2006–2012 (*n* = 75)	2013–2019 (*n* = 91)	2006–2019 (*n* = 152)	2006–2012 (*n* = 63)	2013–2019 (*n* = 89)
Overall survival						
Median, months (95% CI)	109.2 (94.3–NA)	122.4 (100.9–NA)	NA	68.5 (55.5–102.6)	55.5 (31.8–92.1)	NA
Progression-free survival						
Median, months (95% CI)	52.7 (43.5–94.3)	52.7 (43.1–97.4)	52.8 (41.2–NA)	36.9 (29.3–63.3)	28.3 (18.5–55.6)	63.3 (32.0–NA)
Best response to treatment, no. (%)						
ORR	117 (75.5)	54 (75.0)	63 (75.9)	97 (69.0)	37 (63.8)	60 (72.3)
CR or VGPR	35 (22.6)	13 (18.1)	22 (26.5)	25 (17.7)	6 (10.3)	19 (22.9)
PR	59 (38.1)	27 (36.0)	32 (35.2)	42 (29.8)	14 (22.2)	28 (31.5)
MR	23 (14.8)	14 (18.7)	9 (9.9)	30 (21.3)	17 (27.0)	13 (14.6)
SD or PD	38 (24.5)	18 (25.0)	20 (24.1)	44 (31.2)	21 (36.2)	23 (27.7)
Not reported	11 (6.6)	<5	8 (8.8)	11 (7.2)	5 (7.9)	6 (6.7)

1 L: first-line treatment; CI: confidence interval; CR: complete response; MR: minimal response; NA: not available; no.: number; ORR: overall response rate; PR: partial response; PD: progressive disease; SD: stable disease; VA: Department of Veterans Affairs; VGPR: very good partial response.

**Table 6:** Adverse events that resulted in primary treatment discontinuation in veterans with Waldenström macroglobulinemia, 2006 to 2019.

Adverse Events	Younger Population (<70 Years of Age at 1 L Treatment) (*n* = 166)			Older Population (>70 Years of Age at 1 L Treatment) (*n* = 152)		
	2006–2019 (*n* = 166)	2006–2012 (*n* = 75)	2013–2019 (*n* = 91)	2006–2019 (*n* = 152)	2006–2012 (*n* = 63)	2013–2019 (*n* = 89)
Discontinuation due to AE, no. (%)	16 (9.6)	<5	13 (14.3)	30 (19.7)	14 (22.2)	16 (18.0)
1 L discontinued, no./N ( %)						
BDR	<5	0 (0)	<5	<5	<5	<5
BR	<5	0 (0)	<5	5/16 (31.3)	<5	<5
Chloram/FCR/R-CHOP	<5	<5	0 (0)	5/23 (21.7)	5/20 (25.0)	0 (0)
DRC	<5	0 (0)	<5	<5	<5	<5
Ibrutinib +/- R	<5	–	<5	6/17 (35.3)	–	6/17 (35.3)
Other	<5	<5	0 (0)	<5	<5	<5
Single-agent R	<5	0 (0)	<5	6/52 (11.5)	<5	<5

1 L: first-line treatment; AE: adverse event(s); BDR: bortezomib and dexamethasone +/− rituximab; BR: bendamustine +/− rituximab; Chloram: chlorambucil; DRC: dexamethasone, rituximab, and cyclophosphamide; FCR: fludarabine and cyclophosphamide +/− rituximab; no.: number; R: rituximab; R-CHOP: cyclophosphamide, doxorubicin, vincristine, and prednisone ± rituximab.

**Figure A1:**
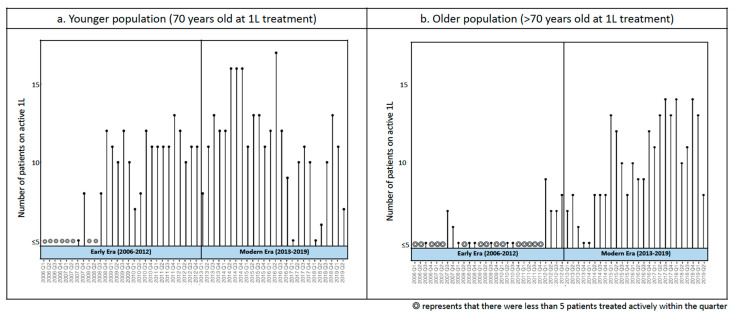
Number of patients receiving active first-line treatments for Waldenström macroglobulinemia per quarter-year, 2006 to 2019: (**a**) number of patients on active first-line treatments in younger population (70 years of age or younger at initiation of 1 L therapy); (**b**) number of patients on active first-line treatments in older population (older than 70 years of age at initiation of 1 L therapy).

## Data Availability

The data underlying this article will be shared upon reasonable request to the corresponding author.
